# Supplementary material for: Exploring the effects of quercetin-added pancreatic diet on metabolic homeostasis in dogs via metabolomics
Source: PLoS One. 2025 Feb 13;20(2):e0318159. doi: 10.1371/journal.pone.0318159 (PMC11824997; doi:10.1371/journal.pone.0318159)
Supplement: Attachment — Submitted filename: Reviewed PONE-D-24-24297.pdf [file pone.0318159.s001.pdf]

PLOS ONE

Exploring the Effects of Quercetin-added Pancreatic Diet on Metabolic Homeostasis in Dogs via Metabolomics

--Manuscript Draft--

**Manuscript Number:**	PONE-D-24-24297
**Article Type:**	Research Article
**Full Title:**	Exploring the Effects of Quercetin-added Pancreatic Diet on Metabolic Homeostasis in Dogs via Metabolomics
**Short Title:**	Quercetin-Enhanced Dog Food for Metabolic Health
**Corresponding Author:**	Juan Wan<br>Gannan Medical University<br>Ganzhou, Jiangxi CHINA
**Keywords:**	canine; pancreatic prescription food; quercetin; metabolic homeostasis; plasma metabolomics
**Abstract:**	Objective<br><br>To investigate the role of quercetin-added pancreatic prescription foodin regulating metabolic homeostasis in dogs.<br><br>Methods<br><br>The experimental dogs were divided into a control diet group and a prescription diet group. The control group was fed regular food, while the prescription group was fed pancreatic prescription food (3.9 g of quercetin was added in per 1 kg of food) for 8 weeks. Canine physical examination, complete blood count, and serum biochemical tests were conducted at 0w, 4w,and 8w. Non-targeted metabolomics tests were performed using plasma samples at 0w and 8w.<br><br>Results<br><br>Dogs that received a quercetin-added pancreatic diet supplemented with quercetin showed significant improvement in metabolic homeostasis, especially in fatty acid, amino acid, and bile acid metabolism. Untargeted metabolomics analysis revealed that quercetin activates ABC transport and arginine/proline pathways, suggesting potential benefits for pancreatitis in large animals, while maintaining comparable safety parameters.<br><br>Conclusions<br><br>Quercetin-added prescription food enhances fatty acid and amino acid metabolism, demonstrating its potential to promote pancreatic function and sustain metabolic homeostasis.
**Order of Authors:**	Juan Wan
	Yaohui Zhang
	Li Xu
	Xiaowan Liu
	Jiabao Xing
	Zhouxiang Wang
	Manli Hu
	Yun Chen
	Zhili Qi
	Yi Ding
	Xin Zhang

Powered by Editorial Manager® and ProduXion Manager® from Aries Systems Corporation

	Mingxing Ding
	Xiaojing Zhang
**Additional Information:**	
**Question**	**Response**
**Financial Disclosure**<br><br>Enter a financial disclosure statement that describes the sources of funding for the work included in this submission. Review the submission guidelines for detailed requirements. View published research articles from *PLOS ONE* for specific examples.<br><br>This statement is required for submission and **will appear in the published article** if the submission is accepted. Please make sure it is accurate.<br><br>**Funded studies**<br>Enter a statement with the following details:<br>• Initials of the authors who received each award<br>• Grant numbers awarded to each author<br>• The full name of each funder<br>• URL of each funder website<br>• Did the sponsors or funders play any role in the study design, data collection and analysis, decision to publish, or preparation of the manuscript?<br><br>Did you receive funding for this work?	Yes
Please add funding details.<br>as follow-up to "**Financial Disclosure**<br><br>Enter a financial disclosure statement that describes the sources of funding for the work included in this submission. Review the submission guidelines for detailed requirements. View published research articles from *PLOS ONE* for specific examples.<br><br>This statement is required for submission and **will appear in the published article** if the submission is accepted. Please make	This work was supported by the National Science Foundation of China (Grant No. 82170595 and 81970070 to X-JZ), the Henan Charity General Federation-Hepatobiliary Foundation of Henan Charity General Federation (Grant No. GDXZ2022008 to XZ, GDXZ2022009 to WJ), Huazhong Agricultural University-Agricultural Genomics Institute at Shenzhen, Cooperation Fund (No. SZYJY2022008 to ZQ), Nationalspecialfundforresearch and development (No. YFD051009 to M-XD).<br><br>Gannan Innovation and Transformation Medical Research Institute, 4# building, PhaseII, high leveltalentsPark, Zhanggonghigh-techzone, Ganzhou City, Jiangxi Province.

sure it is accurate.<br><br>**Funded studies**<br>Enter a statement with the following details:<br>• Initials of the authors who received each award<br>• Grant numbers awarded to each author<br>• The full name of each funder<br>• URL of each funder website<br>• Did the sponsors or funders play any role in the study design, data collection and analysis, decision to publish, or preparation of the manuscript?<br><br>Did you receive funding for this work?"	
Please select the country of your main research funder (please select carefully as in some cases this is used in fee calculation).<br>as follow-up to "**Financial Disclosure**<br><br>Enter a financial disclosure statement that describes the sources of funding for the work included in this submission. Review the submission guidelines for detailed requirements. View published research articles from *PLOS ONE* for specific examples.<br><br>This statement is required for submission and **will appear in the published article** if the submission is accepted. Please make sure it is accurate.<br><br>**Funded studies**<br>Enter a statement with the following details:<br>• Initials of the authors who received each award<br>• Grant numbers awarded to each author<br>• The full name of each funder<br>• URL of each funder website<br>• Did the sponsors or funders play any role in the study design, data collection and analysis, decision to publish, or preparation of the manuscript?<br><br>Did you receive funding for this work?"	CHINA - CN

**Competing Interests**<br><br>Use the instructions below to enter a competing interest statement for this submission. On behalf of all authors, disclose any competing interests that could be perceived to bias this work—acknowledging all financial support and any other relevant financial or non-financial competing interests.<br><br>This statement is required for submission and **will appear in the published article** if the submission is accepted. Please make sure it is accurate and that any funding sources listed in your Funding Information later in the submission form are also declared in your Financial Disclosure statement.<br><br>View published research articles from *PLOS ONE* for specific examples.<br><br>**NO authors have competing interests**<br><br>Enter: *The authors have declared that no competing interests exist.*<br><br>**Authors with competing interests**<br><br>Enter competing interest details beginning with this statement:<br><br>*I have read the journal's policy and the authors of this manuscript have the following competing interests: [insert competing interests here]*<br><br>* typeset	We would like to submit the enclosed manuscript entitled “Exploring the Effects of Quercetin-added Pancreatic Diet on Metabolic Homeostasis in Dogs via Metabolomics”. No conflict of interest exits in the submission of this manuscript, and manuscript is approved by all authors for publication. I would like to declare on behalf of my co-authors that the work described was original research that has not been published previously, and not under consideration for publication elsewhere, in whole or in part. All the authors listed have approved the manuscript that is enclosed.
**Ethics Statement**<br><br>Enter an ethics statement for this submission. This statement is required if the study involved:<br><br>• Human participants<br>• Human specimens or tissue<br>• Vertebrate animals or cephalopods	Ten healthy field dogs were recruited from a clinical practice. The body weight was 8~10 kg, the age was 1~5 years old with no history of disease. Routine in vitro and in vivo deinsectization and vaccine immunization were completed before the trial. This project was approved by the Biomedical Research Ethics Committee of Gannan Medical College.

Powered by Editorial Manager® and ProduXion Manager® from Aries Systems Corporation

• Vertebrate embryos or tissues<br>• Field research<br><br>Write "N/A" if the submission does not require an ethics statement.<br><br>General guidance is provided below. Consult the submission guidelines for detailed instructions. **Make sure that all information entered here is included in the Methods section of the manuscript.**	

Powered by Editorial Manager® and ProduXion Manager® from Aries Systems Corporation

**Format for specific study types**<br><br>**Human Subject Research (involving human participants and/or tissue)**<br>• Give the name of the institutional review board or ethics committee that approved the study<br>• Include the approval number and/or a statement indicating approval of this research<br>• Indicate the form of consent obtained (written/oral) or the reason that consent was not obtained (e.g. the data were analyzed anonymously)<br><br>**Animal Research (involving vertebrate animals, embryos or tissues)**<br>• Provide the name of the Institutional Animal Care and Use Committee (IACUC) or other relevant ethics board that reviewed the study protocol, and indicate whether they approved this research or granted a formal waiver of ethical approval<br>• Include an approval number if one was obtained<br>• If the study involved *non-human primates*, add *additional details* about animal welfare and steps taken to ameliorate suffering<br>• If anesthesia, euthanasia, or any kind of animal sacrifice is part of the study, include briefly which substances and/or methods were applied<br><br>**Field Research**<br><br>Include the following details if this study involves the collection of plant, animal, or other materials from a natural setting:<br>• Field permit number<br>• Name of the institution or relevant body that granted permission	
**Data Availability**<br><br>Authors are required to make all data underlying the findings described fully available, without restriction, and from the time of publication. PLOS allows rare exceptions to address legal and ethical concerns. See the PLOS Data Policy and FAQ for detailed information.	Yes - all data are fully available without restriction

A Data Availability Statement describing where the data can be found is required at submission. Your answers to this question constitute the Data Availability Statement and **will be published in the article**, if accepted.<br><br>**Important:** Stating 'data available on request from the author' is not sufficient. If your data are only available upon request, select 'No' for the first question and explain your exceptional situation in the text box.<br><br>Do the authors confirm that all data underlying the findings described in their manuscript are fully available without restriction?	
**Describe where the data may be found in full sentences. If you are copying our sample text, replace any instances of XXX with the appropriate details.**<br><br>• If the data are **held or will be held in a public repository**, include URLs, accession numbers or DOIs. If this information will only be available after acceptance, indicate this by ticking the box below. For example: *All XXX files are available from the XXX database (accession number(s) XXX, XXX.).*<br>• If the data are all contained **within the manuscript and/or Supporting Information files**, enter the following: *All relevant data are within the manuscript and its Supporting Information files.*<br>• If neither of these applies but you are able to provide **details of access elsewhere**, with or without limitations, please do so. For example:<br><br>*Data cannot be shared publicly because of [XXX]. Data are available from the XXX Institutional Data Access / Ethics Committee (contact via XXX) for researchers who meet the criteria for access to confidential data.*<br><br>*The data underlying the results presented in the study are available from (include the name of the third party*	All relevant data are within the manuscript and its Supporting Information files.

and contact information or URL).<br>• This text is appropriate if the data are owned by a third party and authors do not have permission to share the data.<br><br>* typeset	
Additional data availability information:	

Manuscript

Click here to access/download;Manuscript;Quercetin-added pancreatic prescription.docx

Exploring the Effects of Quercetin-added Pancreatic Diet on Metabolic Homeostasis in Dogs via Metabolomics

Yaohui Zhang[1,2]†, Li Xu[1,3]†, Xiao-Wan Liu[1,3], Jia-Bao Xing[4], Zhouxiang Wang[1,5], Manli Hu[1,6], Yun Chen[1,7], Zhili Qi[8], Yi Ding[9], Xin Zhang[1,6], Ming-Xing Ding[9], Xiao-Jing Zhang[1,5*], Juan Wan[1,6*]

1 Gannan Innovation and Translational Medicine Research Institute; First Affiliated Hospital, Gannan Medical University, Ganzhou, China;

2 School of Basic Medicine, Gannan Medical University, Ganzhou, China;

3 School of Public Health and Health Management, Gannan Medical University, Ganzhou, China;

4 Jiangxi Huichong Technology Co., Ltd., Ganzhou, 341000, China;

5 School of Basic Medicine, Wuhan University, Wuhan, China;

6 Key Laboratory of Prevention and Treatment of Cardiovascular and Cerebrovascular Diseases, Ministry of Education, Gannan Medical University, Ganzhou, China;

7 Huanggang Institute of Translational Medicine, Huanggang, China

8 College of Animal Science and Technology, Huazhong Agricultural University, Wuhan, China

9 College of Veterinary Medicine, Huazhong Agricultural University, Wuhan, China

†These authors contributed equally to this work and share first authorship

*Correspondence to:

wanjuan@gmu.edu.cn (Juan Wan, Ph.D.)

zhangxjing@whu.edu.cn (Xiao-Jing Zhang, Ph.D.)

ABSTRACT

Objective: To investigate the role of quercetin-added pancreatic prescription foodin regulating metabolic homeostasis in dogs. **Methods:** The experimental dogs were divided into a control diet group and a prescription diet group. The control group was fed regular food, while the prescription group was fed pancreatic prescription food (3.9 g of quercetin was added in per 1 kg of food) for 8 weeks. Canine physical examination, complete blood count, and serum biochemical tests were conducted at 0w, 4w,and 8w. Non-targeted metabolomics tests were performed using plasma samples at 0w and 8w. **Results:** Dogs that received a quercetin-added pancreatic diet supplemented with quercetin showed significant improvement in metabolic homeostasis, especially in fatty acid, amino acid, and bile acid metabolism. Untargeted metabolomics analysis revealed that quercetin activates ABC transport and arginine/proline pathways, suggesting potential benefits for pancreatitis in large animals, while maintaining comparable safety parameters. **Conclusions:** Quercetin-added prescription food enhances fatty acid and amino acid metabolism, demonstrating its potential to promote pancreatic function and sustain metabolic homeostasis.

Keywords: canine, pancreatic prescription food, quercetin, metabolic homeostasis, plasma metabolomics

1. INTRODUCTION

Pancreatitis is a potentially devastating, life-threatening condition that affects as many as 80,000 people in the United States annually[1]. Acute pancreatitis is a relatively common clinical condition hallmarked by unregulated trypsin activity within

the pancreatic acinar cell, leading to pancreatic autodigestion and parenchymal inflammation. In contrast, chronic pancreatitis is a progressive fibroinflammatory disease characterized by irreversible loss of the pancreatic parenchyma and subsequent functional insufficiency. Canine pancreatitis has been reported to manifest a similar condition in humans[2]. Dogs with severe acute pancreatitis are typically displayed an acute onset of anorexia, weakness, vomiting, diarrhoea and/or abdominal pain[3, 4]. Chronic pancreatitis in dogs is typically associated with intermittent clinical signs that are less specific and milder than acute pancreatitis[5-7]. Thus, the study of canine pancreatitis helps to address the lack of understanding of its pathophysiological mechanism and provides effective treatment strategies for human pancreatitis.

The development of pancreatitis is the result of various mechanisms, including premature activation of digestive enzymes within pancreatic acinar cells, activation of inflammatory pathways, altered pancreatic microcirculation, oxidative stress, activation of the complement system[8, 9]. Clinical treatment of canine pancreatitis usually contains intravenous injections, antiemetics, analgesia, antibacterial and anti-inflammatory, nutritional supplements and other measures [10]. At present, the incidence of canine pancreatitis is on the rise, leading to increased economic pressure to pet owners[11]. Therefore, it is of significant clinical importance and social value to develop effective pancreatitis treatment drugs for pancreatitis or prescription foods for pets with pancreatic issues.

Quercetin is one of the most widely distributed and most extensively studied flavonoid that are widely present in the human diet. It is found to play a role in

protecting pancreatic related disease, such as pancreatitis, insulin dysfunction, and pancreatic cancer. Quercetin protected against intestinal barrier disruption and inflammation in acute pancreatitis [12]. Quercetin strengthened the survival processes and the secretory capacity of beta cells through several mechanisms, including inhibition of NF-κB signaling, nitric oxide generation, reactive oxygen species levels, increased mitochondrial bioenergetic function, and stimulated pathways of insulin secretion (e.g., PLC/PKC cAMP/PKA, and/or mTOR signaling)[13]. Quercetin supplementation is considered a promising pancreatic-protect compound and to maintain human and other animal health.

In this study, the dogs were fed with an independently developed pancreatic prescription food with quercetin-added, and canine physiological performance, complete blood count, blood biochemical index and plasma metabolomics were investigated to evaluate the regulatory effect of the prescription food on pancreatic function and metabolic homeostasis.

2. MATERIALS AND METHODS

2.1 Prescription food preparation

The formula of canine pancreatic prescription food was designed by referencing the national Standard (GB/T 31216-2014), Full-price Pet Food-Dog Food and American Association of Feed Control Officials (AAFCO), and considering the pathogenesis and nutritional requirements of pancreatitis. The raw materials and additives used in the formula were in line with the provisions of the Feed Raw Materials Catalogue and Feed

Additives Catalogue. The preparation process of prescription food was as follows. Firstly, the raw material with large particles was crushed, sieved and weighed according to the formula, stirred evenly in the mixer. Next, the mixed material was puffed and granulated in the expander. Then the expanded granular material was dried at 65℃ for 4 h to reduce the moisture content to 5%-10%. Finally, the dried prescription food was vacuum bagged. The crude protein, crude fat, crude fiber, ash, moisture, calcium, total phosphorus and energy of the pancreas prescription diet were determined referring to national standards or general method.

2.2 Experimental animals

Ten healthy field dogs were recruited from a clinical practice. The body weight was 8~10 kg, the age was 1~5 years old with no history of disease. Routine in vitro and in vivo deinsectization and vaccine immunization were completed before the trial. This project was approved by the Biomedical Research Ethics Committee of Gannan Medical College. The dogs were randomly divided into control diet group (n=4) and a pancreas prescription diet group (n=6). The control diet group was fed with commercial full price compound food for experimental dogs. The prescription diet group were fed with canine pancreatic prescription food, which was jointly developed by Gannan Institute of Innovation and Translational Medicine and Jiangxi Huichong Technology Co., LTD. All dogs underwent a 2-week food adaptation period before formal feeding. The daily feeding amount was calculated according to the formula: MER /ME, MER = $BW_{kg}^{0.75} \times 70 \times$ life stage factor. The experimental dogs were fed twice a day (at 9:00 am

and 6:00 pm) for 8 weeks. Their daily food intake was recorded during the experiment. Physical examinations including body weight, fasting blood glucose levels, and body condition score (BCS) were performed at 0w, 4w, and 8w. The blood samples were collected for a complete blood count and blood biochemistry examination at 0w, 4w, and 8w. Blood samples at 0w and 8w were also applied metabolically test.

2.3 Physical examination and blood test

The dogs were weighed after fasting overnight, and thier body condition was independently scored by two researchers. Five milliliters of whole blood were collected from the distal cephalic vein. The fasting blood glucose was tested on Blood glucose meter (AccU-Chek Active, Roche， USA) with 20 μL of blood. The blood count was applied on five-category blood count apparatus for animals (Abaxis VetScan HM5, USA) using 1 mL EDTA anticoagulant blood. The remaining blood was stored in a heparin lithium anticoagulant tube and centrifuged at 1500 g for 10 min to obtain upper plasma for biochemical test and metabolomics detection. The biochemical detection was applied on Automatic biochemical analyzer (Hitachi 3110, Japan). Complete blood count and blood biochemical tests were performed by the medical Laboratory of WellAnimal Test (Wuhan) Co., LTD.

2.4 Untargeted metabolomics

The plasma samples for metabolomics detection were transferred to EP tubes and immediately frozen in liquid nitrogen. Before testing, the plasma samples were thawed

slowly at 4°C, and 100 mL of each sample was vortically mixed with 400 mL of pre-cooled methanol/acetonitrile/aqueous solution (2:2:1, V/V). The samples were then ultrasonically treated at 4°C for 30 min, and stood at -20°C for 10 min. After that, the samples were centrifuged at 4°C at 14000 g for 15 min in a low temperature high speed centrifuge (Eppendorf 5430R) to remove the residual proteins, and the supernatant was dried by vacuum. The dried samples were redissolved in acetonitrile solution, and the supernatant was collected by centrifugation for untargeted metabolome sequencing. Untargeted metabolome sequencing was performed by high resolution liquid chromatography (Agilent 1290 Infinity, Agilent, USA) combined with the flight time mass spectrometry (AB Triple TOF 6600, AB Sciex, USA). XCMS software was used for peak alignment, retention time correction and peak area extraction of mass spectrometry, and finally for qualitative and quantitative analysis of metabolites.

2.5 Bioinformatics analysis

Metabolite identification and relative abundance calculations were carried out with metabolomics original results. The metabolite abundance matrix was imported into Ropls software (version No. 1.26.0) for principal component analysis to assess the consistency distribution of samples in different groups. The multivariate statistical OPLS-DA model was used to analyze and screen the metabolites with different abundance. The HMDB database was used to exclude endogenous synthetic metabolites, and then KEGG pathway enrichment analysis was conducted for metabolites with different abundance. Meanwhile, the metabolic spectrum trend of each

KEGG metabolic pathway was determined based on the proportion of all metabolites up- and down-regulated in different groups.

2.6 Statistical analysis

SPSS 21.0 software was used to analyze the difference of blood count and blood biochemical indexes between groups. All data are expressed as mean ± SD. When the data were in line with normal distribution, independent T test was used to compare the difference between the two groups. The non-parametric Mann-Whitney U test was used when the distribution does not conform to a normal distribution. When $P < 0.05$, the difference between groups was considered statistically significant.

3. RESULTS

3.1 Nutrition component of pancreatic prescription food

The crude protein, crude fat, crude fiber, ash, moisture, calcium, total phosphorus and energy values of the food were determined referring to national standards or general method. The detail information of each nutrient component per 100 g of dry matter in both control commercial food and pancreatic prescription food were is presented in **Table 1**. There were 3.1 g of crude fat and 4 g of crude fiber per 100 g dry matter of pancreatic prescription food, while 12.06 g of crude fat and 9 g of crude fiber in that of control food. The added amount of quercetin was 3.6 g per 100 g of dry matter in the pancreatic prescription food. The metabolic energy of the prescription food (3126.16 kcal/kg) is close to control food (3152.09 kcal/kg). These results illustrated that the

formulated pancreatic prescription food has a portrait of low fat, highly digestible and energy balanced.

3.2 Effect of quercetin-added pancreatic prescription diet on physiological indexes and complete blood count in dogs

The experimental dogs were fed with control or quercetin-added pancreatic prescription diet, and showed no abnormality in daily observation. Physical examination, complete blood count and blood biochemistry were performed at 0 w, 4 w and 8 w (**Figure 1A**). The body weight, fasting blood glucose, BCS and other physical indicators of experimental dogs were analyzed and exhibited no difference in the two groups during the experiment (**Figure 1 B-C**). The indexes of whole blood program of red blood cells, white blood cells and platelets showed no change between pancreatic prescription group and control group (**Figure 1D**), which indicates the quercetin-added pancreatic prescription diet has no toxic on blood system.

3.3 Effect of quercetin-added pancreatic prescription diet on blood biochemical indexes in dogs

To fully investigate the safety of the quercetin-added pancreatic prescription diet on key organs, the biochemistry parameters were systematically tested. The lipase levels in plasma were significantly decreased in pancreatic prescription-diet dogs at 8w. No changes were found in the levels of α-amylase between two groups during the experiment. The levels of total protein (TP), albumin (ALB), alanine aminotransferase

(ALT), aspartate aminotransferase (AST), alkaline phosphatase (ALP), triglyceride (TG), total cholesterol (TC), α-amylase (α-AMY), creatine kinase (CK), creatinine (CRE), blood urea nitrogen (BUN), calcium ion (Ca^{2+}), potassium ion (K^{2+}), and sodium ion (Na^{2+}) between pancreatic prescription group and control group showed no obvious difference (**Figure 2**). These biochemical data indicated that the quercetin-added pancreatic prescription diet could promote the pancreatic function, and had no adverse effects on the function of liver, kidney and heart.

3.4 Effects of quercetin-added pancreatic prescription diet on metabolic characteristic in dogs

To analyze the effects of pancreatic prescription diet on the metabolic status of dogs, plasma samples from control-diet and pancreatic prescription-diet dogs at 0 w and 8 w were taken for untargeted metabonomics detection. A total of 1293 plasma metabolites were identified in the untargeted metabolome, of which 675 (52.2%) positive and 433 (33.5%) negative metabolites had clear chemical taxonomy information. By quantitative and proportional analysis, it was found that eight main metabolites were abundant in canine plasma, including lipid and lipid molecules (positive:193 species, 28.5%; negative: 200 species, 46.2%), organic acids and their derivatives (positive:163 species, 24.1%; negative: 88 species, 20.3%), organoheterocyclic compounds (positive: 105 species, 15.6%; negative: 35 species, 8.08%) and others (**Figure 3 A-B**). Principal component analysis (PCA) was used to analyze the overall pattern of plasma metabolic profiles of control diet dogs and pancreatic prescription diet dogs at 0 w and 8 w. It was

found that the plasma metabolic patterns in the two groups were highly overlapped at 0w, after two weeks of adaptive feeding. The overall metabolic characteristics of the control diet dogs were not significantly changed after eight-week feeding (**Figure 3 C-D**). The confidence interval distributions of plasma samples were clearly separated in pancreatic prescription-diet group at 0w and 8w (**Figure 4A**), and in pancreatic prescription-diet group and control-diet group at 8w (**Figure 4B**), indicating there was a significant change in the overall abundance of the blood metabolites. Comparing the total abundance and difference of eight main metabolites in plasma between the two groups at 0w and 8w, the results showed that the abundance of organic nitrogen compounds in the pancreatic prescription diet group was decreased, while the abundance of organic oxygen compounds, phenylpropane and polykeones was increased at 8w (**Figure 4C**).

3.5 Quercetin-added pancreatic prescription diet regulates pancreatic function through pentose phosphate pathway and others

After PCA analysis of plasma metabolic profiles in control and pancreatic prescription diet dogs at 0 w and 8 w, the OPLS-DA model was applied to screen the characteristic differential metabolites. The results of model replacement test showed that the model interpretation rate (R2) and prediction ability (Q2) of the random model gradually decreased with the decrease of replacement retention, indicating that the model has good robustness and can be used for feature selection and modeling. Subsequently, model VIP > 1 and threshold $P < 0.05$ of inter-group T-test were used to screen

metabolites with characteristic differences in pancreatic prescription-diet group at 0w and 8w (**Figure 5A-B**). The same model was used to screen the characteristic differential metabolites in pancreatic prescription-diet group and control-diet group at 8w (**Figure 5C-D**). The KEGG annotation was performed for the metabolites with different abundance to calculate the enrichment degree of each pathway. The results showed that the metabolites with different abundance were mainly concentrated in ABC transporter, arginine and proline metabolism, tyrosine metabolism, unsaturated fatty acid synthesis and other pathways (**Figure 5E-F**). Combined with the overall change trend of metabolites in each enrichment pathway in the pancreatic prescription-diet group at 0w and 8w, the absolute value of differential abundance scores was set as greater than two times threshold. it was found that the abundance of metabolites in ABC transporters, arginine and proline metabolism, tyrosine metabolism, primary bile acid biosynthesis, pentose phosphate pathway, pentose and glucuronate interconversions, and alanine, aspartate and glutamate metabolism were increased. While unsaturated fatty acids were decreased (**Figure 5G**). The changes of key differential metabolites in the above significantly regulated metabolic pathways were shown in **Figure 5H**.

4. DISCUSSION

The canine pancreatitis is mainly caused by unhealthy diet habits. Obese dogs are more prone to pancreatitis, frequently after eating a lot of fatty food [14, 15]. The occurrence and development of canine pancreatitis are associated with the pathological mechanisms of pancreatic microcirculation disorder, cascade effect of cytokines,

systemic inflammatory response, pancreatic cell apoptosis and intestinal bacterial migration [16]. The dogs with pancreatitis are generally displayed depressed, anorexia and vomiting. The treatment of pancreatitis should be considered to remove the cause, maintain fluid and electrolyte balance and other support therapy. Treatment for complications could promote recovery of affected dogs [2]. The pancreatic prescription food adding edible Chinese medicine ingredients to enhance pancreatic function effectively ameliorate pancreatitis.

Previous studies indicated that prolonged consumption of diets rich in fats may trigger pancreatitis [17-19]. The digest function was found to be damaged in pancreatitis-attacked patients and animals. The pancreatic prescription diet with reasonable ratio of various nutritional composition, such as low fat and highly digestible, is a new adjuvant therapying approach in clinical. In the present study, we independently developed a pancreatic prescription food with quercetin supplement for dogs. the formulated pancreatic prescription food of low fat and highly digestible is conformed to the nutrition demand for pancreatitis-attacked dogs. During the experiment, all dogs could eat up the food they received and showed no abnormality in daily observation, suggestion the quercetin-added pancreatic prescription diet is a well tasting and safe food.

After 8-week feeding, the quercetin-added pancreatic prescription diet group showed a significant decrease in plasma lipase levels, which is a relative specific

indicator of pancreatic function and a key feature of pancreatic function recovery[20]. Pancreatic lipase breaks down the majority of dietary triacylglycerols in the gastrointestinal tract, thereby significantly contributing to the absorption of lipids, inhibiting the lipid metabolism-related inflammatory cascades[21]. The increased abundance of pentose phosphate pathway in dogs with pancreatic prescription diet is associated with the ability of anti-inflammation. The pentose phosphate pathway converts glucose-6-phosphate into pentoses and generates ribose-5-phosphate and NADPH thereby governing anabolic biosynthesis and redox homeostasis, generate fuels NOX2 to reduce reactive oxygen species which ameliorate the inflammatory response [22], the non-oxidative pentose phosphate pathway was reported to regulate regulatory T function to prevent autoimmunity [23].

Natural plant additives were often added into diets as functional active substance. Quercetin, a flavonoid compound with various biological activities, is widely found in a variety of plants (ginkgo, notoginseng, buckwheat, elderberry, guava, etc.) and food (such as asparagus, cabbage, tea, grapes, apples, etc.). Quercetin has a wide range of biological effects. Quercetin could inhibit the growth, migration, and invasion and induced apoptosis of pancreatic cancer cells by antagonizing SHH and TGF-β/Smad signaling pathways [24]. Studies showed that quercetin down-regulated the expression and activation of NF-κB, a key inflammatory factor, in the progression of pancreatitis [2]. Previous studies reported that quercetin intervention exerted therapeutic effects on pancreatitis, intestinal injury and inflammation by inhibiting the activation of

TLR4/MyD88/P38 MAPK pathway by up-regulating miR-216b and endoplasmic reticulum stress [25, 26]. Quercetin could modulate NADPH oxidase-derived $O_2^{\cdot -}$ production in macrophages, suppress NADPH oxidase-dependent oxidative stress to exert anti-oxidant and anti-inflammatory properties [27]. In this study, quercetin-added pancreatic prescription diet promoted pancreatic and maintain metabolic homeostasis, properly via restraining NADPH oxidase-dependent oxidative stress related to pentose phosphate pathway. However, the regulated mechanism needs further research. Whatever, the results of the present study provide evidence for treatment strategy for human pancreatitis.

Quercetin, as a common feed plant additive, is widely used in the development of functional fodder. Quercetin supplemented to the diet can improve immune function and the quality of egg in aging hens, regulate gut microbial metabolism so as to attenuate diarrhea in piglets [28-30]. In the present study, dogs feeding with quercetin-added pancreatic prescription displayed improved the pancreatic function during long-term feeding, suggesting that the quercetin-added prescription diet has a good prospect for development.

5. CONCLUSION

In conclusion, quercetin-added pancreatic prescription diet can promote pancreatic function and metabolic homeostasis via increasing pentose phosphate pathway metabolism. Our study provides new insight into molecular mechanisms of quercetin-

added pancreatic prescription on therapying pancreatitis.

6. CONFLICT OF INTEREST

Author J-BX were employed by Jiangxi Huichong Technology Co., Ltd (Ganzhou, China). The remaining authors declare that the research was conducted in the absence of any commercial or financial relationships that could be construed as a potential conflict of interest.

Reference

1. Marshall, S.M., *The pancreas in health and in diabetes.* Diabetologia, 2020. **63**(10): p. 1962-1965.
2. Cridge, H., et al., *Advances in the diagnosis of acute pancreatitis in dogs.* J Vet Intern Med, 2021. **35**(6): p. 2572-2587.
3. Weatherton, L.K. and E.M. Streeter, *Evaluation of fresh frozen plasma administration in dogs with pancreatitis: 77 cases (1995-2005).* J Vet Emerg Crit Care (San Antonio), 2009. **19**(6): p. 617-22.
4. Watson, P., *Pancreatitis in dogs and cats: definitions and pathophysiology.* J Small Anim Pract, 2015. **56**(1): p. 3-12.
5. Bostrom, B.M., et al., *Chronic pancreatitis in dogs: a retrospective study of clinical, clinicopathological, and histopathological findings in 61 cases.* Vet J, 2013. **195**(1): p. 73-9.
6. Xenoulis, P.G., *Diagnosis of pancreatitis in dogs and cats.* J Small Anim Pract, 2015. **56**(1): p. 13-26.

7. Watson, P.J., et al., *Observational study of 14 cases of chronic pancreatitis in dogs.* Vet Rec, 2010. **167**(25): p. 968-76.

8. Gukovskaya, A.S., et al., *Autophagy, Inflammation, and Immune Dysfunction in the Pathogenesis of Pancreatitis.* Gastroenterology, 2017. **153**(5): p. 1212-1226.

9. Tokoro, T., et al., *Interactions Between Neutrophils and Platelets in the Progression of Acute Pancreatitis.* Pancreas, 2020. **49**(6): p. 830-836.

10. Mansfield, C. and T. Beths, *Management of acute pancreatitis in dogs: a critical appraisal with focus on feeding and analgesia.* J Small Anim Pract, 2015. **56**(1): p. 27-39.

11. Gori, E., et al., *Acute pancreatitis and acute kidney injury in dogs.* Vet J, 2019. **245**: p. 77-81.

12. Carvalho, K.M., et al., *The natural flavonoid quercetin ameliorates cerulein-induced acute pancreatitis in mice.* Biol Pharm Bull, 2010. **33**(9): p. 1534-9.

13. Eid, H.M. and P.S. Haddad, *The Antidiabetic Potential of Quercetin: Underlying Mechanisms.* Curr Med Chem, 2017. **24**(4): p. 355-364.

14. Xenoulis, P.G. and J.M. Steiner, *Canine hyperlipidaemia.* J Small Anim Pract, 2015. **56**(10): p. 595-605.

15. Qu, W., et al., *Profound Perturbation in the Metabolome of a Canine Obesity and Metabolic Disorder Model.* Front Endocrinol (Lausanne), 2022. **13**: p. 849060.

16. Holowaychuk, M.K., *Hypocalcemia of critical illness in dogs and cats.* Vet Clin North Am Small Anim Pract, 2013. **43**(6): p. 1299-317, vi-vii.

17. Thomas, T., L. Mah, and S.G. Barreto, *Systematic review of diet in the pathogenesis of acute pancreatitis: a tale of too much or too little?* Saudi J Gastroenterol, 2012. **18**(5): p. 310-5.

18. Navina, S., et al., *Lipotoxicity causes multisystem organ failure and exacerbates acute*

pancreatitis in obesity. Sci Transl Med, 2011. **3**(107): p. 107ra110.

19. Nawaz, H., et al., *Elevated serum triglycerides are independently associated with persistent organ failure in acute pancreatitis.* Am J Gastroenterol, 2015. **110**(10): p. 1497-503.

20. Cridge, H., et al., *Association between abdominal ultrasound findings, the specific canine pancreatic lipase assay, clinical severity indices, and clinical diagnosis in dogs with pancreatitis.* J Vet Intern Med, 2020. **34**(2): p. 636-643.

21. Toldi, V., N. Kassay, and A. Szabó, *Missense PNLIP mutations impeding pancreatic lipase secretion cause protein misfolding and endoplasmic reticulum stress.* Pancreatology, 2021. **21**(7): p. 1317-1325.

22. Amara, N., et al., *Selective activation of PFKL suppresses the phagocytic oxidative burst.* Cell, 2021. **184**(17): p. 4480-4494.e15.

23. Liu, Q., et al., *Non-oxidative pentose phosphate pathway controls regulatory T cell function by integrating metabolism and epigenetics.* Nat Metab, 2022. **4**(5): p. 559-574.

24. Guo, Y., et al., *Quercetin suppresses pancreatic ductal adenocarcinoma progression via inhibition of SHH and TGF-β/Smad signaling pathways.* Cell Biol Toxicol, 2021. **37**(3): p. 479-496.

25. Sheng, B., et al., *Quercetin inhibits caerulein-induced acute pancreatitis through regulating miR-216b by targeting MAP2K6 and NEAT1.* Inflammopharmacology, 2021. **29**(2): p. 549-559.

26. Junyuan, Z., et al., *Quercetin protects against intestinal barrier disruption and inflammation in acute necrotizing pancreatitis through TLR4/MyD88/p38 MAPK and ERS inhibition.* Pancreatology, 2018. **18**(7): p. 742-752.

27. Luo, M., et al., *Quercetin suppressed NADPH oxidase-derived oxidative stress via heme*

oxygenase-1 induction in macrophages. Arch Biochem Biophys, 2019. **671**: p. 69-76.
28. Amevor, F.K., et al., *Synergistic effects of quercetin and vitamin E on egg production, egg quality, and immunity in aging breeder hens.* Poult Sci, 2021. **100**(12): p. 101481.
29. Zoidis, E., et al., *Quercetin and Egg Metallome.* Antioxidants (Basel), 2021. **10**(1).
30. Xu, B., et al., *Dietary Quercetin Supplementation Attenuates Diarrhea and Intestinal Damage by Regulating Gut Microbiota in Weanling Piglets.* Oxid Med Cell Longev, 2021. **2021**: p. 6221012.

Table

Table 1: Nutritional components of control diet and pancreatic prescription diet

Nutritional components	Control diet	Prescription diet
Crude protein（g）	18.95	21.48
Crude ash（g）	9.61	6.1
Crude fat（g）	12.06	3.1
Crude fibre（g）	9	4
water（g）	7.65	10
Total Carbohydrate（g）	51.73	59.32
Quercetin（g）	0	3.6
Metabolic energy（kcal/kg）	3152.09	3126.16

Figure Legends

Figure 1. The analysis of physiological indexes and complete blood count in dogs

fed with quercetin-added pancreatic prescription diet

(A) Schematic diagram of the experimental design process. Dogs were randomly divided into two groups, and then fed with a quercetin-added pancreatic prescription diet or a control diet for 8 weeks after a 2-week adaptation period, respectively. The dogs were evaluated by physical examination, laboratorial examination at 0w, 4w and 8w.

(B) Analysis of body weight, body condition score (BCS) and fasting blood glucose in the prescription diet group and control diet group at 0w, 4w and 8w. n=6 in prescription diet group, and n=4 in control diet group.

(C) Whole blood parameter analysis of dogs fed with prescription diet and control diet. n=6 in prescription diet group, and n=4 in control diet group.

RBC, red blood cell; WBC, white blood cell; LYM, lymphocytes; MON, monocytes; NEU, neutrophils; PLT, platelets; MPV, mean platelet volume; RDWs, red cell distribution width; MCV, mean corpuscular volume; PDWs, platelet distribution width ; HGB, hemoglobin; MCHC, mean corpuscular hemoglobin concentration; MCH, mean corpuscular hemoglobin; RBC%, the percent of red blood cell in blood cells; PLT%, the percent of platelets in blood cells.

n.s. represents a P value greater than 0.05 (not statistically significant)

Figure 2. The analysis of blood biochemical indexes in dogs fed with quercetin-added pancreatic prescription diet

TP, total protein; ALB, albumin; ALT, alanine aminotransferase; AST, aspartate

aminotransferase; ALP, alkaline phosphatase; TG, triglyceride; TC, total cholesterol; α-AMY, α-amylase; CK, creatine kinase; CREA, creatinine; BUN, blood urea nitrogen; Ca^{2+}, calcium ion; K^{+}, potassium ion; Na^{+}, sodium ion.

* represents a P value less than 0.05, n.s. represents a P value greater than 0.05 (not statistically significant), compared with the control diet group. Statistical analysis was carried out by Student's two-tailed t test.

Figure 3. Untargeted metabolomics analysis of canine plasma

(A, B) Statistics of all identifified metabolites according to the attribution information of chemical taxonomy. The specifific number (A) and the proportion (B) of each metabolite superclass in plasma samples are presented, respectively.

(C, D) Principal component analysis (PCA) of plasma samples in control diet group and prescription diet group at 0w (C), and in control diet group at 0w and 8w (D) in positive (left) and negative (right) ion modes.

Figure 4. The metabolic characteristic of plasma in dogs fed with quercetin-added pancreatic prescription diet

(A, B) Principal component analysis (PCA) of plasma samples in prescription diet group at 0w and 8w (A), and in control diet group and prescription diet group at 8w (B) in positive (left) and negative (right) ion modes.

(C) The comparsion of the total abundance and difference of main metabolites in plasma between control diet group and prescription diet group at 0w and 8w.

* represents a P value less than 0.05, n.s. represents a P value greater than 0.05 (not statistically significant), compared with the control diet group. Statistical analysis was carried out by Student's two-tailed t test.

Figure 5. Differential abundance metabolites screening and downstream pathway analysis of quercetin-added pancreatic prescription diet

(A, B) Orthogonal partial least squares discriminant analysis (OPLS-DA) model of canine plasma samples in the prescription diet group at 0w and 8w (A), and screening of differential metabolites based on the variable importance for the projection (VIP) in the OPLS-DA model (B) in positive (left) and negative (right) ion modes. Significant difference was defined as VIP > 1 and $P < 0.05$.

(C, D) OPLS-DA of canine plasma samples in the control diet group and prescription diet group at 8w (C), and screening of differential metabolites based on the VIP in the OPLS-DA model (D) in positive (left) and negative (right) ion modes. Signifificant difference was defined as VIP > 1 and $P < 0.05$.

(E, F, G) Kyoto Encyclopedia of Genes and Genomes (KEGG) pathway enrichment analysis of differential metabolites in plasma of prescription-diet dogs at 0w and 8w, and of control-diet and prescription-diet dogs at 8w.

(H) The endogenous differential metabolites with the highest fold change of expression after merging positive and negative ion patterns.

手稿

Click here to access/download;Manuscript;Quercetin-added pancreatic prescription.pdf

Exploring the Effects of Quercetin-added Pancreatic Diet on Metabolic Homeostasis in Dogs via Metabolomics

Yaohui Zhang[1,2]†, Li Xu[1,3]†, Xiao-Wan Liu[1,3], Jia-Bao Xing[4], Zhouxiang Wang[1,5], Manli Hu[1,6], Yun Chen[1,7], Zhili Qi[8], Yi Ding[9], Xin Zhang[1,6], Ming-Xing Ding[9], Xiao-Jing Zhang[1,5]*, Juan Wan[1,6]*

1 Gannan Innovation and Translational Medicine Research Institute; First Affiliated Hospital, Gannan Medical University, Ganzhou, China;

2 School of Basic Medicine, Gannan Medical University, Ganzhou, China;

3 School of Public Health and Health Management, Gannan Medical University, Ganzhou, China;

4 Jiangxi Huichong Technology Co., Ltd., Ganzhou, 341000, China;

5 School of Basic Medicine, Wuhan University, Wuhan, China;

6 Key Laboratory of Prevention and Treatment of Cardiovascular and Cerebrovascular Diseases, Ministry of Education, Gannan Medical University, Ganzhou, China;

7 Huanggang Institute of Translational Medicine, Huanggang, China

8 College of Animal Science and Technology, Huazhong Agricultural University, Wuhan, China

9 College of Veterinary Medicine, Huazhong Agricultural University, Wuhan, China
