# Supplementary material for: Exploring the effects of quercetin-added pancreatic diet on metabolic homeostasis in dogs via metabolomics
Source: PLoS One. 2025 Feb 13;20(2):e0318159. doi: 10.1371/journal.pone.0318159 (PMC11824997; doi:10.1371/journal.pone.0318159)
Supplement: Attachment — Submitted filename: Review comments.pdf [file pone.0318159.s002.pdf]

**Comments and Suggestions for Authors**

**The study titled "Exploring the Effects of Quercetin-Enhanced Pancreatic Diet on Metabolic Homeostasis in Dogs Using Metabolomics," authored by Yaohui Zhang et al., investigates the role of a quercetin-enriched pancreatic prescription diet in regulating metabolic homeostasis in dogs, with a particular focus on fatty acid, amino acid, and bile acid metabolism. Utilizing advanced techniques such as untargeted metabolomics and bioinformatics analysis, the research examines the diet's impact on key metabolic pathways. The findings suggest that long-term administration of a quercetin-enhanced diet may yield significant and lasting benefits for pancreatic health in dogs. This study highlights the diet's potential to support pancreatic function and promote metabolic homeostasis, offering valuable insights into the therapeutic potential of quercetin for managing pancreatitis. The manuscript is well-written. The reviewer has only some minor concerns as follows：**

**1. In the Materials and Methods section, the study was conducted with a limited sample size of 10 dogs, comprising 6 in the experimental group and 4 in the control group. This small sample size may limit the generalizability and statistical power of the results. The authors may wish to discuss additional factors that influenced the decision to use a smaller sample size, such as ethical considerations related to animal welfare or other relevant constraints.**

**2. In this study, authors mentioned in abstract and that untargeted metabolomics analysis revealed that quercetin activates ABC transport and arginine/proline pathways, suggesting potential benefits for pancreatitis in large animals. However, author may need to discuss more detail or analyze current metabolomics data to clarify the role of ABC transport on promoted pancreatic function and sustain metabolic homeostasis after animal fed quercetin added food.**

**3. In Figure 3, the Principal Component Analysis (PCA) revealed overlapping confidence intervals in some instances, suggesting that the metabolic profiles between groups may not be as distinct as initially claimed. The authors might consider discussing this observation more detail in the Results or Discussion section.**

**4. While the study demonstrates that quercetin influences metabolic pathways such as ABC transporters and the pentose phosphate pathway, the authors could use the current data to provide a more detailed mechanistic explanation of how quercetin exerts these effects. The absence of this analysis leaves an incomplete understanding of quercetin's role. The authors may wish to address this in the Discussion section.**
