# Supplementary material for: Exploring the effects of quercetin-added pancreatic diet on metabolic homeostasis in dogs via metabolomics
Source: PLoS One. 2025 Feb 13;20(2):e0318159. doi: 10.1371/journal.pone.0318159 (PMC11824997; doi:10.1371/journal.pone.0318159)
Supplement: Attachment — Submitted filename: response to reviewers.docx [file pone.0318159.s003.docx]

Dear Editors and Reviewer,

Thank you very much for your critical comments and thoughtful suggestions. We have made careful modification on the original manuscript. All changes made to the text are in red in the revised manuscript so that they made be easily identified. All the questions are answered below.

**Response to Reviewer 1:**

**Q1. In the Materials and Methods section, the study was conducted with a limited sample size of 10 dogs, comprising 6 in the experimental group and 4 in the control group. This small sample size may limit the generalizability and statistical power of the**

**results. The authors may wish to discuss additional factors that influenced the decision to use a smaller sample size, such as ethical considerations related to animal welfare or other relevant constraints.**

**Response 1:** We acknowledge this good suggestion. We added “In consideration of the principle of minimal animal use for animal welfare, 4 dogs in the control group and 6 dogs in the experimental group were used in this study. Although individual differences within groups were minimal, the limited sample size in this study may still affect the statistical power of the results.” in Discussion section in Line 316-319.

**Q2. In this study, authors mentioned in abstract and that untargeted metabolomics**

**analysis revealed that quercetin activates ABC transport and arginine/proline**

**pathways, suggesting potential benefits for pancreatitis in large animals. However,**

**author may need to discuss more detail or analyze current metabolomics data to clarify**

**the role of ABC transport on promoted pancreatic function and sustain metabolic homeostasis after animal fed quercetin added food.**

**Response 2:** Thanks for your comments. The abundance of metabolites in ABC transporters in quercetin-added pancreatic prescription diet group were increased. ABC transporters reduced inflammatory signaling pathways in atherosclerosis (Fitzgerald ML, Mujawar Z, Tamehiro N. ABC transporters, atherosclerosis and inflammation. Atherosclerosis. 2010 Aug;211(2):361-70. doi: 10.1016/j.atherosclerosis.2010.01.011), and reglated the development and function of different T cell populations(Thurm C, Schraven B, Kahlfuss S. ABC Transporters in T Cell-Mediated Physiological and Pathological Immune Responses. Int J Mol Sci. 2021 Aug 25;22(17):9186. doi: 10.3390/ijms22179186). ABC transporters are one of the better known mechanisms of drug resistance in cancer (Gu, J., Huang, W., Wang, X. et al. Hsa-miR-3178/RhoB/PI3K/Akt, a novel signaling pathway regulates ABC transporters to reverse gemcitabine resistance in pancreatic cancer. Mol Cancer 21, 112 (2022). [https://doi.org/10.1186/s12943-022-01587-9; El-Mahdy HA, El-Husseiny AA, Kandil YI, Gamal El-Din AM. Diltiazem potentiates the cytotoxicity of gemcitabine and 5-fluorouracil in PANC-1 human pancreatic cancer cells through inhibition of P-glycoprotein. Life Sci. 2020;262:118518).](https://doi.org/10.1186/s12943-022-01587-9).) Chen et al. reported that quercetin could inhibit the expression levels of FZD7, ABC transporters (ABCB1, ABCC1 and ABCC2) and beta-catenin in hepatocellular carcinoma cells (Chen Z, Huang C, Ma T, Jiang L, Tang L, Shi T, Zhang S, Zhang L, Zhu P, Li J, Shen A. Reversal effect of quercetin on multidrug resistance via FZD7/β-catenin pathway in hepatocellular carcinoma cells. Phytomedicine. 2018 Apr 1;43:37-45. doi: 10.1016/j.phymed.2018.03.040). Quercetin-added pancreatic prescription diet in this diet played a role in pancreatic protection probably through modulating metabolic homeostasis by promoting ABC transporters and pentose phosphate pathway.

The related content has been added to the Discussion section in Line 368-370.

**Q3. In Figure 3, the Principal Component Analysis (PCA) revealed overlapping**

**confidence intervals in some instances, suggesting that the metabolic profiles between**

**groups may not be as distinct as initially claimed. The authors might consider discussing this observation more detail in the Results or Discussion section.**

**Response 3:** Thanks for your suggestions. We have added “The above results indicate that all dogs showed no significant differences in metabolite levels prior to enrollment, and the control diet had minimal impact on their metabolite composition” in the Result section in Line 273-275.

**Q4. While the study demonstrates that quercetin influences metabolic pathways such as ABC transporters and the pentose phosphate pathway, the authors could use the**

**current data to provide a more detailed mechanistic explanation of how quercetin**

**exerts these effects. The absence of this analysis leaves an incomplete understanding of quercetin's role. The authors may wish to address this in the Discussion section.**

**Response 4:** We sincerely thank the reviewer for good suggestion. The pentose phosphate pathway is divided into branches that produce the nucleotide precursor ribose-5-phosphate and the cofactor NADPH, which serves as a reducing agent during the biosynthesis of lipids and amino acids (Stincone A, Prigione A, Cramer T, Wamelink MM, Campbell K, Cheung E, Olin-Sandoval V, Grüning NM, Krüger A, Tauqeer Alam M, Keller MA, Breitenbach M, Brindle KM, Rabinowitz JD, Ralser M. The return of metabolism: biochemistry and physiology of the pentose phosphate pathway. Biol Rev Camb Philos Soc. 2015 Aug;90(3):927-63. doi: 10.1111/brv.12140). Quercetin could modulate NADPH oxidase-derived O_2_^.-^ production in macrophages, suppress NADPH oxidase-dependent oxidative stress to exert anti-oxidant and anti-inflammatory properties. In this study, quercetin-added pancreatic prescription diet promoted pancreatic and maintain metabolic homeostasis, properly via restraining NADPH oxidase-dependent oxidative stress related to pentose phosphate pathway.

The related content has been added to the Discussion section in Line 383-391.

**Response to Reviewer 2:**

**Q1: Initiate with the information on canine physical examination results is strongly advocated.**

**Response 1:** We sincerely appreciate the valuable comments. We have added the results of the body weight, fasting blood glucose, BCS, the indexes of whole blood program, and the blood biochemical indexes in the Abstract. Please check the details in Line 53-61.

**Q2: For better paper structure, the methods to measure variables are categorized according to the following:**

**1. Canine physical examination,**

**2. Complete blood count and serum biochemical tests**

**3. Untargeted metabolomics analysis**

**Aligned with the information in the abstract.**

**Response 2:** Thanks for your critical comments. According to your suggestion, we have adjusted the method structure in the revised manuscript.

**Q3: refocused on the measured variables:**

**1. Canine physical examination,**

**2. Complete blood count and serum biochemical tests**

**3. Untargeted metabolomics analysis**

**Then, compare the findings data with references.**

**Response 3:** As suggested by the reviewer, we have added the discussion about canine physical indexes, the indexes of whole blood program, and the blood biochemical indexes, and compared our data with references. The related content are in the Discussion section in Line 361-370.
